# Prioritization of zoonoses for multisectoral, One Health collaboration in Somalia, 2023

**DOI:** 10.1016/j.onehlt.2023.100634

**Published:** 2023-09-22

**Authors:** Abdinasir Yusuf Osman, Halima Mohamed, Farah I. Mumin, Heba Mahrous, Asma Saidouni, Sharifo Ali Elmi, Amira Khalif Adawe, Abdikani Abdullahi Mo'allim, Mutaawe Lubogo, Sk Md Mamunur Rahman Malik, Athman Mwatondo, Tajudeen Raji, Abdifatah Dirie Ahmed, Alimuddin Zumla, Osman Dar, Richard Kock, Siobhan M. Mor

**Affiliations:** a Royal Veterinary College, University of London, London, UK; b National Institute of Health, Ministry of Health, Mogadishu, Somalia; c Wolfson Institute of Population Health, Queen Mary University of London, London, UK; d Institute of Infection, Veterinary and Ecological Sciences, University of Liverpool, Neston, UK; e International Livestock Research Institute, Addis Ababa, Ethiopia; f Red Sea University, Bosaso, Somalia; g World Health Organization, Regional Office for the Eastern Mediterranean, Cairo, Egypt; h Ministry of Livestock Forestry and Range, Mogadishu, Somalia; i Faculty of Veterinary Medicine, University Malaysia Kelantan, Kelantan, Malaysia; j Ministry of Environment and Climate Change, Mogadishu, Somalia; k Ministry of Agriculture and Irrigation, Mogadishu, Somalia; l World Health Organization, Country Office, Mogadishu, Somalia; m Zoonotic Disease Unit, Ministry of Health, Nairobi, Kenya; n Africa Centres for Disease Control and Prevention, Addis Ababa, Ethiopia; o National Institute for Health and Care Research Biomedical Research Centre, University College London Hospitals NHS Foundation Trust, London, UK; p Department of Infection, Division of Infection and Immunity, University College London, London, UK; q Global Health Programme, Royal Institute of International Affairs, London, UK; r Global Operations, United Kingdom Health Security Agency, London, UK

**Keywords:** Zoonotic diseases, One Health, Somalia, Prioritization

## Abstract

**Background:**

The human population of Somalia is vulnerable to zoonoses due to a high reliance on animal husbandry. This disease risk is exacerbated by relatively low income (poverty) and weak state capacity for health service delivery in the country as well as climate extremes and geopolitical instability in the region. To address this threat to public health efficiently and effectively, it is essential that all sectors have a common understanding of the priority zoonotic diseases of greatest concern to the country.

**Methods:**

Representatives from human, animal (domestic and wildlife), agriculture, and environmental health sectors undertook a multisectoral prioritization exercise using the One Health Zoonotic Disease Prioritization (OHZDP) tool developed by the United States CDC. The process involved: reviewing available literature and creating a longlist of zoonotic diseases for potential inclusion; developing and weighting criteria for establishing the importance of each zoonoses; formulating categorical questions (indicators) for each criteria; scoring each disease according to the criteria; and finally ranking the diseases based on the final score. Participants then brainstormed and suggested strategic action plans to prevent, and control prioritized zoonotic diseases.

**Results:**

Thirty-three zoonoses were initially considered for prioritization. Final criteria for ranking included: 1) socioeconomic impact (including sensitivity) in Somalia; 2) burden of disease in humans in Somalia); 3) availability of intervention in Somalia; 4) environmental factors/determinants; and 5) burden of disease in animals in Somalia. Following scoring of each zoonotic disease against these criteria, and further discussion of the OHZDP tool outputs, seven priority zoonoses were identified for Somalia: Rift Valley fever, Middle East respiratory syndrome, anthrax, trypanosomiasis, brucellosis, zoonotic enteric parasites (including *Giardia* and *Cryptosporidium*), and zoonotic influenza viruses.

**Conclusions:**

The final list of seven priority zoonotic diseases will serve as a foundation for strengthening One Health approaches for disease prevention and control in Somalia. It will be used to: shape improved multisectoral linkages for integrated surveillance systems and laboratory networks for improved human, animal, and environmental health; establish multisectoral public health emergency preparedness and response plans using One Health approaches; and enhance workforce capacity to prevent, control and respond to priority zoonotic diseases.

## 1 Introduction

According to the latest World Health Organization (WHO) definition, a zoonosis is any disease or infection that is naturally transmissible from vertebrate animals to humans [1]. Most (60%) existing human infectious diseases and at least 70% of (re-)emerging infectious diseases of humans have an animal origin [2,3]. Out of five new human diseases reported on average every year, three are of animal origin and the majority (80%) of agents with potential bioterrorist use are zoonotic pathogens [4]. The apparent failure of global health security to prevent or prepare for the COVID-19 pandemic and its likely origin in animals has highlighted the weakness in public health systems and the need for closer cooperation between human, animal (domestic and wildlife), and environmental health [5].

Located in the Horn of Africa, Somalia occupies the tip of a region that is particularly challenging for epidemic control. Weak health systems, shortage of trained health workers, frequent extreme weather events such as droughts and flooding, relatively low national income, informal maritime transport networks, and geopolitical instability all contribute to this challenge and increase potential for infectious disease spread. Of an estimated 16.88 million people in Somalia, around one half (8.25 million people) require humanitarian assistance and protection [6]. The country is particularly vulnerable to zoonotic diseases due to a high reliance on animal husbandry for sustenance and livelihood [7]. Pastoralists make up around 55% of Somalia's population [8]. Regular movement of people and livestock is integral to livelihoods and social cohesion in pastoralist communities and is highly influenced by climate events [9]. Most of these movements occur across borders with Ethiopia and Kenya, increasing the threat of disease introduction to/from neighboring countries. The problem of zoonoses is exacerbated in Somalia by weak state capacity for implementing border controls for animal and human populations [10], along with presence of terrorist groups inside the country with stated intent to use biological weapons (Gen. Mohamed Mohamud Garar, personal communication, 20 June 2023).

One Health programming in Somalia has been limited to date, partly because the country has not been a beneficiary of many of the One Health initiatives in the region. Nonetheless, the National Institute of Health (NIH), Ministry of Health, Somalia, in collaboration with relevant federal line ministries, do advocate for the institutionalization and operationalization of One Health in the country. By linking humans, animals and the environment, One Health can help to address the full spectrum of disease control – from prevention to detection, preparedness, response and management – and contribute to global health security [11].

During the joint external evaluation (JEE) conducted in Somalia in 2015, anthrax, brucellosis, bovine tuberculosis, Rift Valley fever (RVF) and toxoplasmosis were stated as priority zoonotic diseases [12]. However, the methodology and process used to derive that list has not been published. Recently, an updated list was generated following a One Health Zoonotic Disease Prioritization (OHZDP) workshop. The goal of the workshop was to use a multisectoral, One Health approach to prioritize zoonoses of greatest concern and develop next steps and action plans to address the priority diseases in collaboration with One Health partners. In this paper, we report on the process and outcomes of the workshop.

## 2 Methods

### 2.1 Workshop organization and participant selection

The OHZDP workshop for Somalia was held in Nairobi, Kenya, between February 7–9, 2023. The core planning team for the workshop consisted of 10 human, animal, agricultural and environmental health experts from the Federal Government of Somalia. The OHZDP workshop was supported financially and technically by the University of Liverpool under the auspices of the GCRF-funded HORN project (https://onehealthhorn.net/). Lead facilitation was provided by the World Health Organization Regional Office for the Eastern Mediterranean (WHO-EMRO). To build in-country capacity to conduct future OHZDP workshops, WHO-EMRO staff trained eight local partners to serve as facilitators in the days preceding the workshop (February 5–6, 2023).

Participants for the workshop were drawn from federal member state governments representing the human, animal, agricultural and environmental health sectors in Somalia; academia (including Somali nationals working in international universities); local and regional non-governmental organizations (NGOs); and key international and supra-international organizations. Participants were divided into voting members, facilitators, and advisors based on their expertise and responsibilities assigned in the workshop. The 12 voting members were equally represented by human, animal, agriculture, and environmental health sectors. All voting members were technical leaders or managers working in Somali line ministries or local academic institutions.

### 2.2 Literature review and initial list of zoonotic diseases

The workshop used the mixed methods prioritization tool developed by the US Centers for Disease Control and Prevention's (CDC) One Health Office. The methods have been previously described in detail [13]. An initial list of diseases was developed following a systematic review conducted by the authors prior the workshop. The systematic review is published in the same issue of this journal [14]. Briefly, we searched Web of Science to identify research studies on zoonotic diseases in Somalia. The initial list of search terms was developed using knowledge of the diseases present in Somalia. Given the recognized limited research output on zoonoses in Somalia [15], these keywords were supplemented by the names of diseases included in OHZDP lists from other countries in the region, particularly Ethiopia, Kenya, and Uganda [[16], [17], [18]]. For purposes of workshop preparation, a further search of documents from websites of governmental, and other relevant organizations including WHO, World Organisation for Animal Health (WOAH, founded as OIE), and ProMED was undertaken. Data on disease transmission, severity, pandemic and epidemic potential, economic impact, prevention and control, and environmental impact were captured for each zoonotic disease in a spreadsheet. In total, data from 76 research articles and other sources were collated and shared with the core planning team for review and consideration. At this stage, *Giardia* was added given its perceived contribution to childhood diarrhea. Consideration was also given to adding COVID-19 but this was omitted as the impacts of the pandemic had largely passed. The final list presented for endorsement in the workshop included 33 diseases. This list included diseases that are acquired by humans principally through zoonotic transmission (e.g., brucellosis); diseases with a zoonotic origin but where the primary mode of transmission is human-to-human (e.g., Ebola); diseases with known zoonotic and non-zoonotic transmission pathways in East Africa (e.g., trypanosomiasis); and diseases with an unclear but feasible zoonotic component based on findings from elsewhere (e.g., chikungunya, dengue).

### 2.3 Criteria selection

During the workshop, participants jointly developed five criteria for ranking the 33 diseases. Once the five criteria were agreed, advisors developed one categorical question for each criterion through group discussion. The questions were developed to best measure each criterion, taking into consideration the availability of evidence from Somalia and the region. All questions had ordinal, binomial or multinomial answers. The ordinal nature was necessary for the scoring process. Each answer choice was given a score, which was determined by the advisors during the question development process. Simultaneously, the voting members individually ranked their preferences for the relative importance of each of the five criteria. Each individual voting member's ranking was inputted into the OHZDP tool by a facilitator and a group weight calculated for each criterion.

### 2.4 Disease scoring and final ranking

Working in facilitated groups, advisors went through each of the 33 diseases on the list and answered each categorical question using data in the spreadsheet generated through the literature review. Where information for a particular zoonotic disease was not available for Somalia specifically, expert knowledge and data from the region or globally were used to inform the answers. For each disease, responses to each question were inputted into the OHZDP tool [13]. The tool then automatically calculated the weighted score for each criterion for each disease. Subsequently a sum of scores for each disease was generated and normalized so that the highest final score was one. The ranked list of zoonotic diseases and corresponding normalized scores were presented to the 12 voters to discuss and decide on a final list of priority zoonotic diseases for Somalia.

### 2.5 Next steps and action plans

After finalizing the list of priority zoonotic diseases, workshop representatives were divided into five groups to discuss next steps, action plans and approaches to effectively address the agreed priority zoonotic diseases for Somalia using a multisectoral, One Health approach. Major thematic areas discussed included multisectoral coordination mechanism (MCM); preparedness planning; surveillance; strengthening laboratory systems and networks to ensure early detection; and workforce capacity.

## 3 Results

### 3.1 Participants

The OHZDP workshop for Somalia had a total of 53 participants including facilitators (*n* = 12), advisors (*n* = 29) and voters (*n* = 12). The organizational affiliation of participants is shown in Table 1. The Federal Government of Somalia was represented by senior experts from the Ministry of Health; Ministry of Livestock, Forestry and Range; Ministry of Agriculture; and Ministry of Environment and Climate Change. Five federal member states were represented in the workshop and included the primary ecological and socio-demographic regions of Galmudug, Hirshabelle, Jubaland, Puntland, and Southwest region. Provinces in Somaliland were not able to attend however local academic and NGO managers from Somaliland participated in the workshop and provided input.

**Table 1:** Participating organizations in the One Health Zoonotic Disease Prioritization workshop for Somalia, February 5–9, 2023. Note: Some participants had multiple affiliations.

**Voting Members**
*Federal Government of Somalia*
Ministry of Health (2)
National Institute of Health (1)
Ministry of Environment and Climate Change (2)
Ministry of Livestock, Forestry and Range (1)
Ministry of Agriculture and Irrigation (3)
*Federal Member States*
Ministry of Environment and Climate Change, Puntland State (1)
Ministry of Livestock, Forestry and Range, Jubaland State (1)
*Academia*
Abrar University, Somalia (1)
**Advisors**
*Government*
Ministry of Health, Somalia
National Institute of Health, Somalia
Ministry of Environment and Climate Change, Somalia
Ministry of Livestock, Forestry and Range, Somalia
Ministry of Agriculture and Irrigation, Somalia
Ministry of Rural Development and Resilience, Somalia
Somali Bureau of Standards, Somalia
*Academia*
Amoud University, Somaliland, Somalia
Arizona State University, USA
Heritage Institute for Policy Studies, Somalia
Queen Marry University of London, UK
Red Sea University, Puntland, Somalia
Royal Veterinary College, University of London, UK
Somali National University, Mogadishu, Somalia
University College London, UK
*NGOs and key international and supra-international organizations*
Deutsche Gesellschaft für Internationale Zusammenarbeit (GIZ)
Food and Agriculture Organization (FAO)
Global Implementation Solutions (GIS)
International Livestock Research Institute (ILRI)
Vétérinaires Sans Frontières Suisse (VSF-Suisse)
World Health Organization Regional Office for the Eastern Mediterranean (WHO-EMRO)
World Health Organization Somalia Country Office
World Organisation for Animal Health (WOAH, formerly OIE)
**Facilitators**
*Government*
Ministry of Health, Somalia
National Institute of Health, Somalia
Ministry of Livestock, Forestry and Range, Somalia
*Academia*
Red Sea University, Puntland, Somalia
Universiti Malaysia Kelantan, Malaysia
Royal Veterinary College, University of London, UK
University of Liverpool, UK
*NGOs and key international and supra-international organizations*
International Livestock Research Institute (ILRI)
UK Health Security Agency (UKHSA)
World Health Organization Regional Office for the Eastern Mediterranean (WHO-EMRO)
World Health Organization Somalia Country Office

### 3.2 Endorsement of the list of diseases

Following presentation of the initial list of 33 diseases, some discussion followed on the potential omission of cholera and fish-borne diseases. These diseases were regarded as relevant and important examples of One Health disease-related problems in the country. However, owing to the focus of the workshop on zoonoses specifically, and the unclear zoonotic significance of the diseases in question in Somalia, the 12 voting members agreed to endorse the list of 33 diseases as presented for prioritization.

### 3.3 Criteria and question development

The five agreed criteria and their associated weights are given in Table 2**.** The criteria and phrasing of the questions were chosen because they emphasized the local importance of livestock-generated livelihoods and vulnerability of the country to climate-related disasters whilst also recognizing the limitations in data availability for Somalia.

**Table 2:** Final criteria, criterion weights and associated questions used for ranking zoonotic diseases using the One Health Zoonotic Disease Prioritization (OHZDP) tool during the workshop for Somalia, February 5–9, 2023.

Rank	Criteria	Weight	Question	Answer (score)
1	Socio-economic impact (including sensitivity)	0.43	What is the socio-economic, political and security impact of the disease?	Minimal impact (0) Low impact (1) Moderate impact (2) High impact (3) Very high impact (4)
2	Burden of disease in humans (endemicity/impact on human health)	0.16	Has the disease been detected/occurred in Somalia or East Africa region in the last 10 years in humans?	No, not in Somalia or East Africa (0) Yes, in the region (East Africa) only (1) Yes, in Somalia only (2) Yes, in Somalia and East Africa (3)
3	Availability of intervention (feasibility)	0.16	Are control measures available in Somalia?	No, not available for humans or animals (0) Yes, available in humans only (1) Yes, available in animals only (2) Yes, available for both humans and animals (3)
4	Environmental factors/ determinants (transmissibility)	0.13	Is increased incidence/prevalence of the disease and the impact of the environment associated with extreme weather events?	No (0)Yes, weak (1)Yes, moderate (2)Yes, strong (3)
5	Burden of disease in animals (endemicity/impact on animal health)	0.12	Has the disease been detected/occurred in Somalia or the East Africa region within the last 10 years in animals?	No, not in Somalia or East Africa (0) Yes, in the region (East Africa) only (1) Yes, in Somalia only (2) Yes, in Somalia and East Africa (3)

### 3.4 Final ranking of diseases

The final scores and ranking of diseases is presented in Table 3. Upon review and discussion of the rankings, the 12 voting members decided to combine giardiasis (ranked 6th) and cryptosporidiosis (ranked 26th) under the designation of ‘zoonotic enteric parasites’. Subsequently, the voting members reached agreement on a final list of seven priority zoonotic diseases for Somalia. This included (from highest to lowest priority): Rift Valley fever, Middle East respiratory syndrome (MERS), anthrax, trypanosomiasis, brucellosis, zoonotic enteric parasites and zoonotic influenza viruses. Although the normalized final score for zoonotic influenza viruses was 0.303 (ranked 27th), it was included as the 7th priority disease following discussions amongst the voting members who perceived that the disease would attract greater external donor funding.

**Table 3:** Raw and normalized scores and ranks of 33 diseases generated using the One Health Zoonotic Disease Prioritization (OHZDP) tool during the workshop for Somalia, February 5–9, 2023.

Rank	Zoonosis or Zoonotic Origin Disease	Raw Score	Normalized Score
1	Rift Valley fever	0.839	1
2	Middle East respiratory syndrome	0.816	0.973
3	Anthrax	0.814	0.970
4	Trypanosomiasis	0.687	0.819
5	Brucellosis	0.654	0.780
6	Giardiasis	0.598	0.713
7	Rabies	0.546	0.651
8	Fascioliasis (liver fluke)	0.546	0.651
9	Echinococcosis (hydatidosis)	0.546	0.651
10	Toxoplasmosis	0.546	0.651
11	Campylobacteriosis	0.511	0.609
12	Salmonellosis	0.511	0.609
13	Bovine tuberculosis	0.492	0.587
14	Ebola virus	0.46	0.548
15	Chikungunya virus	0.453	0.540
16	Dengue fever	0.453	0.540
17	Leptospirosis	0.447	0.533
18	Crimean-Congo hemorrhagic fever	0.44	0.525
19	Q fever	0.42	0.500
20	Schistosomiasis	0.398	0.474
21	Leishmaniasis	0.393	0.469
22	Rickettsiosis	0.364	0.434
23	*E. coli*	0.35	0.417
24	Camelpox	0.332	0.395
25	Cysticercosis/Taeniasis	0.331	0.395
26	Cryptosporidiosis	0.296	0.353
27	Zoonotic influenza viruses	0.255	0.303
28	Listeriosis	0.252	0.301
29	Hepatitis E	0.248	0.295
30	Orf (contagious ecthyma)	0.185	0.220
31	Aspergillosis	0.159	0.190
32	Yellow fever	0.14	0.167
33	West Nile virus	0.039	0.047

### 3.5 Next steps and action plans

Recommendations for next steps and action plans are presented in the supplementary material.

## 4 Discussion

The OHZDP workshop for Somalia generated a consensus on the seven priority zoonotic diseases in the country. The priority zoonoses, as agreed by the multisectoral group of national and international experts during the OHZDP workshop were Rift Valley fever, MERS, anthrax, trypanosomiasis, brucellosis, zoonotic enteric parasites (including *Giardia* and *Cryptosporidium*), and zoonotic influenza viruses. This list includes five endemic and/or epidemic-prone zoonoses known to be historically present in Somalia (Rift Valley fever, anthrax, trypanosomiasis, brucellosis, zoonotic enteric parasites), and two emerging, zoonotic-origin diseases where presence in Somalia has not been clearly established but where an outbreak in humans would be considered significant (MERS and zoonotic influenza viruses).

The methodology developed by CDC – which is intended to be grounded on published literature specific to the country [13] – proved quite challenging to implement in Somalia, where the prevailing conditions have hindered zoonoses surveillance, reporting and research. In a recent scoping review of zoonoses research, Somalia ranked 5/7 countries in the Horn of Africa in terms of number of publications on the subject matter, followed only by the much smaller countries of Eritrea and Djibouti [15]. Consequently, selecting criteria for ranking and scoring of each zoonotic disease based on empirical data (e.g., incidence, prevalence, and mortality) proved challenging in this workshop. Weighing and scoring criteria were mainly influenced by the known and potential social, economic, political, and security impacts on human and animal populations in the region, rather than disease burden data. Further, although the OHZDP tool generated scores and ranks for each disease using country-specific ranking criteria and questions, during the workshop the voting members brought a motion to elevate the ranking of zoonotic influenza viruses. The inclusion of non-endemic diseases – such as zoonotic influenza viruses – in priority lists of many low and middle income countries (LMICs) including Somalia suggest the process may be reactive to the interest and availability of resources by external donors despite these diseases having a minimal disease burden (in terms of morbidity, mortality, and prevalence) or substantial economic impact in these countries [[19], [20], [21]]. As a fragile state Somalia is particularly sensitive to the funding priorities of external donors given that over 90% of its health budget comes from these sources [22].

Of the seven diseases prioritized during the workshop for Somalia, three diseases (Rift Valley fever, brucellosis, and anthrax) were ranked in the top five zoonoses using the CDC tool in Ethiopia (2019 update, [27]), Kenya [16] and Uganda [17]. Zoonotic influenza viruses were also ranked in the top five diseases in Ethiopia, whereas they were ranked in the top ten diseases in Kenya and Uganda. Trypanosomiasis ranked second and sixth in Kenya and Uganda, respectively. Notably, rabies was not in the top five diseases in Somalia, although it ranked highly in neighboring Ethiopia and Kenya. According to the WHO rabies dashboard, Somalia is endemic for rabies but no data on human rabies deaths is available [23]. To our knowledge only one published study attempted to diagnose rabies in Somalia (in wild caught animals) and all samples were negative [24]. In any case, the limited importance of rabies in Somalia was supported by expert opinion in the OHZDP workshop. MERS was considered in lists for Ethiopia and Kenya however it did not score highly in final rankings. The high ranking of MERS in the Somalia workshop may relate to the fact that the workshop was conducted in a post-COVID era, where heightened significance was attached to the pandemic potential of zoonotic coronaviruses. Although MERS only sporadically transmits from camels to humans experts at the workshop were cognizant of the potential for significant, negative impacts of trade restrictions on the camel-raising population in Somalia should the virus acquire enhanced capacity for human-to-human transmission [25].

Since the workshop there has been discussion on the need to consider bovine tuberculosis (ranked 13th in the OHZDP workshop) as a priority zoonosis given perceived importance in Somalia and the greater region [26]. Further consideration has also been given to prioritizing Ebola (ranked 14th in the workshop) given extensive travel links between Somalia and Uganda, the latter country having troops stationed inside Somalia and having experienced several historical outbreaks of this disease. Accordingly, it is now proposed that all nine diseases (seven diseases prioritized in the meeting plus bovine tuberculosis and Ebola) should be included and reported in a National Notifiable Disease Reporting System under a designated National One Health entity or authority for human, animal, plant, and environmental health-related infections. Although some diseases were already notifiable to Somalia's human and animal health agencies, during the workshop participants acknowledged that the disease-specific variation in diagnostic testing capacities of existing laboratory systems and policies across sectors impeded national and state-level control, prevention, preparedness, and response efforts in Somalia. Nonetheless, it was recognized that minimum capacity does exist for laboratory confirmation of all prioritized zoonotic diseases (including viral, bacterial, and parasitic ones) in both humans and animals in Somalia.

Given high rates of migration in the Horn of Africa, workshop discussions emphasized the importance of cross-border collaboration and partnership building for One Health in Somalia. During the workshop, disease experts from other countries, including Kenya and Ethiopia, shared helpful insights on experiences with zoonosis prioritization and subsequent design of surveillance and response systems including the need to harmonize data collection (including standardization) across all relevant sectors. Significant emphasis was also placed on the need for stakeholder engagement and partnership building across relevant sectors and creating regional, national, and local networks and task forces. To this end, it was recognized that there was a need to establish and/or re-invigorate platforms for multisectoral coordination at the national and federal state level to aid in preparedness planning and response efforts, including mobilizing resources for case investigation and detection, and sharing information across sectors during outbreaks and other emergencies.

Since the workshop the Ministry of Health, Federal Republic of Somalia has announced the establishment of the One Health National-Level Technical Working Group (OHNL-TWG) which will provide guidance on the development of a long-term strategic approach to reducing the risk of zoonotic pandemics, and institutionalizing and implementing the One Health approach, including in areas that drive pandemic risk, in the country. Such efforts will help Somalia and the region to better prepare for and respond to future zoonotic disease pandemics, as well as endemic zoonoses which impact on the health and wealth of Somalia's pastoralist populations.

## Author contributions

AYO conceptualized, developed the outline for the article and produced the first draft of the manuscript. SMM, FIM, RK, HM, AZ, and OD edited and contributed to several drafts of the manuscript. All authors contributed to the writing and finalization of the manuscript.

## Funding

The OHZDP workshop was funded by an award to SMM (PI) from a UK Research and Innovation (UKRI) block grant to the University of Liverpool which aimed to increase impact of the One Health Regional Network for the Horn of Africa (HORN) Project. HORN was supported by the Global Challenges Research Fund (GCRF) through a grant from UKRI and 10.13039/501100000268Biotechnology and Biological Sciences Research Council (BBSRC) (project number BB/P027954/1).

## Declaration of Competing Interest

The authors declare the following financial interests/personal relationships which may be considered as potential competing interests:

SMM is on the editorial board for the journal, One Health.

## Data Availability

The authors confirm that the data supporting the findings of this study are available within the article and/or its supplementary materials.

## Appendix A Supplementary data

Supplementary material Image 1
