# Supplementary material for: Prioritization of zoonoses for multisectoral, One Health collaboration in Somalia, 2023
Source: One Health. 2023 Sep 22;17:100634. doi: 10.1016/j.onehlt.2023.100634 (PMC10665150; doi:10.1016/j.onehlt.2023.100634)
Supplement: Supplementary file 1 — Supplementary material [file mmc1.pdf]

ONE HEALTH

One Health Zoonotic Disease Prioritization Process

Prioritizing Zoonotic Diseases for Multisectoral, One Health Collaboration in Somalia

Workshop Summary (abbreviated)

Note: names and photos have been withheld for publication purposes.

EXECUTIVE SUMMARY

The purpose of the One Health Zoonotic Disease Prioritization workshop for Somalia was to prioritize zoonotic diseases of greatest concern using a multisectoral, One Health approach with equal input from representatives of human, animal (livestock and wildlife), and environmental health sectors and other relevant partners.

The specific workshop goals were to use a multisectoral, One Health approach to

1. Prioritize zoonotic diseases of greatest concern
2. Develop next steps and action plans to address the priority zoonotic diseases in collaboration with One Health partners

During the workshop, participants developed a list of zoonotic diseases for prioritization for Somalia, defined the criteria for prioritization, and determined questions and weights relevant to each criterion. A total of 6 zoonotic diseases were identified as a priority by a subset of voting participants (12) using a mixed methods prioritization process, the One Health Zoonotic Disease Prioritization Process, developed by the U.S. Centers for Disease Control and Prevention (CDC) (Appendix A).

After the participants selected the priority zoonotic diseases, participants developed next steps and action plans to address the priority zoonotic diseases in collaboration with One Health partners.

The priority zoonotic diseases for multisectoral, One Health collaboration for Somalia are shown below:

- Rift Valley fever
- Middle East respiratory syndrome
- Anthrax
- Trypanosomiasis
- Brucellosis
- Zoonotic enteric parasites (including *Giardia* and *Cryptosporidium*)
- Zoonotic influenza viruses

This report summarizes the One Health Zoonotic Disease Prioritization process used to prioritize zoonotic diseases of greatest concern for Somalia, as well as next steps and action plans to jointly address these zoonotic diseases using a multisectoral, One Health approach including human, animal, and environmental health ministries, and other relevant sectors.

Background

Somalia is bordered by Kenya to the southwest, the Gulf of Aden to the north, the Guardafui Channel and Indian Ocean to the east, and Ethiopia to the west. Strategically located at the mouth of the Bab el Mandeb gateway to the Red Sea and the Suez Canal, the country occupies the tip of a region that, due to its resemblance on the map to a rhinoceros' horn, is commonly referred to as the Horn of Africa. Somalia has the longest coastline on the mainland of Africa, with a seaboard that stretches 3,333 kilometres (2,071 mi). Its terrain consists mainly of plateaus, plains and highlands [1].

Agriculture Profile

Agriculture is the most important economic sector of Somalia. It accounts for about 65% of the GDP and employs 65% of the workforce. Livestock contributes about 40% to GDP and more than 50% of export earnings [2]. Other principal exports include fish, charcoal and bananas; sugar, sorghum and corn are products for the domestic market [3]. Primarily as a result of substantial local investment by the Somali diaspora, many of these small-scale plants have re-opened and newer ones have been created. The latter include fish-canning and meat-processing plants in the northern regions, as well as about 25 factories in the Mogadishu area, which manufacture pasta, mineral water, confections, plastic bags, fabric, hides and skins, detergent and soap, aluminium, foam mattresses and pillows, fishing boats, carry out packaging, and stone processing [4].

Environment Profile

Somalia is a semi-arid country with about 1.64% arable land. Owing to Somalia's proximity to the equator, there is not much seasonal variation in its climate [5]. Hot conditions prevail year-round along with periodic monsoon winds and irregular rainfall [6]. Somalia contains a variety of mammals and birds due to its geographical and climatic diversity [7]. Currently, there are remnant and threatened populations of various wild herbivores, including antelope, equids, suids, and carnivores, small mammals, primates, many bird, reptile and amphibian species [8, 9]. It also has a large, domesticated population of the dromedary camel [10]. Following the massive tsunami of December 2004, there have also emerged allegations that after the outbreak of the Somali Civil War in the late 1980s, Somalia's long, remote shoreline was used as a dump site for the disposal of toxic waste [11, 12]. The huge waves that battered northern Somalia after the tsunami are believed to have stirred up tons of nuclear and toxic waste that might have been dumped illegally in the country by foreign firms. According to reports by the United Nations Environment Programme (UNEP), the waste has resulted in far higher than normal cases of respiratory infections, mouth ulcers and bleeding, abdominal haemorrhages and unusual skin infections among many inhabitants of the areas around the northeastern towns of Hobyo and Benadir on the Indian Ocean coast diseases consistent with radiation sickness [11, 13]. UNEP adds that the situation along the Somali coastline poses a very serious environmental hazard not only in Somalia, but also in the eastern Africa sub-region [11, 14].

Health Profile

Several decades of civil war have diminished Somalia's health system and displaced 2.6 million people within the country. The current drought situation in Somalia, which has surpassed the 2010-11 and 2016-17 droughts in terms of duration and severity, continues to worsen. Five consecutive failed rainy seasons have affected 8.7 million people with over 1.9 million displaced [15, 16]. The onset of the ongoing rainy season was largely delayed, and the upcoming March-May rainy season is also expected to be poor, humanitarian needs will continue increasing well into 2023. Hunger levels continued to rise, with children affected the most. The population of Somalia is estimated to be 16.88 million based on the latest United Nations data for 2022, of which about 17.8% are children under 5 years. An estimated 8.2 million people in the country require humanitarian assistance and protection.

Somalia One Health Approach

One Health is an integrated, unifying approach that recognizes that the health of humans, domestic and wild animals, plants, and the wider environment (including ecosystems) are closely linked and interdependent. While health, food, water, energy, and environment are all wider topics with sector-specific concerns, the collaboration across sectors and disciplines contributes to protect health, address health challenges such as the emergence of infectious diseases, antimicrobial resistance, and food safety and promote the health and integrity of our ecosystems. By linking humans, animals and the environment, One Health can help to address the full spectrum of disease control – from prevention to detection, preparedness, response and management – and contribute to global health security [17]. The approach can be applied at the community, subnational, national, regional, and global levels, and relies on shared and effective governance, communication, collaboration and coordination. Having the One Health approach in place makes it easier for people to better understand the co-benefits, risks, trade-offs and opportunities to advance equitable and holistic solutions.

INTRODUCTION

Zoonotic diseases are caused by pathogens that originate in animals and infect humans either by direct natural transmission from animal reservoirs (zoonoses) or through spill over and adaptation resulting in novel human pathogens which subsequently spread amongst humans. Around 60 percent of human pathogens have animal origins and around 75% of recently emergent human pathogens also follow this pathway. Only about 15% of human pathogens are ongoing zoonoses (where the infection is normally acquired directly from animals). Some pathogens acquired zoonotically or as regular zoonoses are also pathogenic to animals including rabies, but many are not.

These zoonotic diseases that occur in large numbers can impact society in three main ways. Specifically, they:

- Threaten the health of animals resulting in illness, loss of productivity, and death.
- Threaten the livelihood of the population dependent on livestock as a major source of income.
- Threaten the health of people, with ability to cause illness and death, which is associated with significant social and economic losses.

In order to best address zoonotic disease threats, a multisectoral, One Health approach is needed. One Health means a collaborative, multisectoral, and transdisciplinary approach—working at the local, regional, national, and global levels—with the goal of achieving optimal health outcomes recognizing the interconnection between people, animals, plants, and their shared environment.

To begin addressing zoonotic disease challenges in Somalia, a One Health Zoonotic Disease Prioritization workshop was held on 7-9 February 2023, at the Ole Sereni Hotel in Nairobi, Kenya. The purpose of the One Health Zoonotic Disease Prioritization workshop for Somalia was to prioritize zoonotic diseases of greatest concern using a multisectoral, One Health approach with equal input from representatives of human, animal (livestock and wildlife), and environmental health sectors and other relevant partners.

The specific workshop goals were to use a multisectoral, One Health approach to

1. Prioritize zoonotic diseases of greatest concern
2. Develop next steps and action plans to address the priority zoonotic diseases in collaboration with One Health partners

To build in-country capacity to conduct future One Health Zoonotic Disease Prioritization workshops, 8 local partners were trained by WHO EMRO on the One Health Zoonotic Disease Prioritization process on February 5-6, 2023, from the following relevant One Health sectors and partners:

- Ministry of Health
- National Institute of Health

- Ministry of Environment and Climate Change
- Ministry of Livestock, Forestry and Range
- Ministry of Agriculture and Irrigation
- Red Sea University

Detailed information related to organizational affiliations of participants is given in Table 2.

TABLE 2: PARTICIPATING ORGANIZATIONS IN THE SOMALI ONE HEALTH ZOONOTIC DISEASE PRIORITIZATION WORKSHOP IN NAIROBI, KENYA, FEBRUARY 5–9, 2023

Voting Members (No. participants)
National Level
Ministry of Health (2)
National Institute of Health (1)
Ministry of Environment and Climate Change (2)
Ministry of Livestock, Forestry and Range (1)
Ministry of Agriculture and Irrigation (3)
State level
Ministry of Environment and Climate Change, Puntland State (1)
Ministry of Livestock, Forestry and Range, Jubaland State (1)
Academia
Abrar university (1)
Facilitators
Ministry of Health
National Institute of Health
Ministry of Livestock, Forestry and Range
WHO Regional Office for the Eastern Mediterranean
WHO, Country Office, Somalia
The UK Health Security Agency
Royal Veterinary College, University of London, UK
Red Sea University
International Livestock Research Institute
University of Liverpool
Advisors and Other Participants
Ministry of Health
National Institute of Health
Ministry of Environment and Climate Change
Ministry of Livestock, Forestry and Range
Ministry of Agriculture and Irrigation
Ministry of Rural Development and Resilience
Somali Bureau of Standards
Somali National University
Amoud University
Heritage Institute
Red Sea University
Arizona State University

Royal Veterinary College, University of London, UK
Queen Marry University of London
University College London
University of Liverpool
International Livestock Research Institute
WHO Regional Office for the Eastern Mediterranean
WHO, Country Office, Somalia
World Organisation for Animal Health (OIE)
Food and Agriculture Organization (FAO)
Global Implementation Solutions (GIS)
Vétérinaires Sans Frontières Suisse
GIZ

WORKSHOP METHODS

The One Health Zoonotic Disease Prioritization process uses a mixed methods prioritization process developed by the U.S. Centers for Disease Control and Prevention's (CDC) One Health Office. The methods have been previously described in detail (Appendix A). Workshop organizers began to prepare and plan for this workshop months in advance. During the workshop, participants first reviewed the initial zoonotic disease list to focus on for prioritization Zoonotic diseases on human or animal reportable disease lists were included on the initial list. A list of 33 zoonotic diseases, shown in Appendix C, were considered during the workshop.

During the workshop, participants developed five criteria for ranking the 33 zoonotic diseases of which a few were endemic zoonoses eg., Rift Valley Fever (RVF). Some of the diseases were zoonotic origin pathogens of concern to human health including MERS coronavirus as they may establish and spread within human populations after spill over. Once the five criteria were developed, one categorical question was developed for each criterion through group discussion. The questions were developed to best measure each criteria. All questions had ordinal, binomial or multinomial answers. The ordinal nature is necessary for the scoring process and each answer choice was given a score, which was determined by the participants. Voting members then individually ranked their preferences for the relative importance of each criterion. Each individual voting member's ranking were then inputted into the One Health Zoonotic Disease Prioritization Tool by a facilitator and a group weight for each criteria was calculated. Facilitators and participants answered each question for each zoonotic disease using data that were identified through an extensive literature search, as well as information from WHO, OIE, ProMED, and other relevant websites. Data on disease transmission, severity, pandemic and epidemic potential, economic impact, prevention and control, and environmental impact were collected for each zoonotic disease. If there is relatively little documentation in or from Somalia of zoonoses and zoonotic origin diseases from the list, data from the region and globally were used as proxy. Over 77 articles were collected with zoonotic disease-specific information on transmission, severity, pandemic and epidemic potential, economic impact, prevention and control, and environmental impact for the country, region, and globally. These references were compiled and shared with all workshop participants.

After scoring all zoonotic diseases, decision tree analysis was used to determine the ranked zoonotic disease list. Each weighted criterion was applied across each question's answers for each zoonotic disease. The scores for all five questions for each zoonotic disease were summed. The largest raw score was then normalized giving that zoonotic disease a normalized score of 1. See Appendix C for a complete listing of raw and normalized scores for all zoonotic diseases that were considered for prioritization.

The zoonotic diseases with their raw and normalized scores were presented to the participants for discussion. Workshop participants then utilized the ranked OHZDP list to discuss and decide on a final priority list of 7 zoonotic diseases (Table 1). After the participants decided on the priority zoonotic diseases, participants developed next steps and action plans to address the priority zoonotic diseases.

CRITERIA AND QUESTION DESCRIPTION DEVELOPED

The criteria for ranking zoonotic diseases selected by the voting members in Somalia are listed in order of importance below. A description of how the questions assessed the criteria are listed below. For the full question and answer choices, see Appendix D.

Rank	Criteria	Weight	Question Description
1	**Socioeconomic Impact (including sensitivity)**	0.43	What is the Socio-Economic, political and security impact of the disease?
2	**Burden of disease in humans**	0.16	Is there infection prevalence in the Somalia and East Africa region in the last 10 years in humans?
3	**Availability of Intervention (feasibility)**	0.16	Are there disease control measures available in the country?
4	**Environmental Aspects**	0.13	Is increased incidence/Prevalence of the disease and the impact of the environment is associated with extreme weather events?
5	**Burden of disease in animals**	0.12	Has the disease been detected/occurred in Somalia and in the East Africa region within the last 10 years in animals?

PRIORITY ZOONOTIC DISEASE LIST FOR SOMALIA

According to this workshop outcome, the 7 priority potentially zoonotic diseases for multisectoral, One Health collaboration for Somalia are (Table 1 A). Epidemiologic data for some of the prioritized diseases is given in Table 1 B.

**Table 1A: Priority zoonotic diseases selected in Somalia by subset of voting participants (n=12) in the One Health Zoonotic Disease Prioritization workshop conducted 7-9 February 2023**
1. Rift Valley fever
2. Middle East respiratory syndrome
3. Anthrax
4. Trypanosomiasis
5. Brucellosis
6. Zoonotic enteric parasites (including *Giardia* and *Cryptosporidium*)
7. Zoonotic influenza viruses

Table 1. Epidemiological data of priority zoonotic diseases selected in Somalia by subset of voting participants (n=12) in the One Health Zoonotic Disease Prioritization workshop conducted 7-9 February 2023

Zoonotic Disease	Agent	Prevalence/Incidence/Outbreaks in Human	Prevalence/Incidence/Outbreaks in Animals	Diagnostics, Treatment & Prevention in Place
Rift Valley fever	RVF virus	Between Dec 2006 to Feb 2007, 51 deaths (case–fatality rate, 45%) were reported to WHO from Somalia's Southern region (WHO, 2007)	An overall 0.3% of all cattle, sheep and goats studied (Hassan-Kadle, Osman et al. 2021). sero-prevalence of 2% (90/4570) and 5% (206/4050) in Somaliland and Puntland respectively (Soumare et al. 2007)	A national contingency plan for Rift Valley Fever developed in 2020
Middle East Respiratory Syndrome	Middle East Respiratory Syndrome Coronavirus	N/A	N/A	Neither treatment nor diagnostic plan is in place
Anthrax	Bacillus anthracis	N/A	In 2011, the annual report from AU-IBAR showed that Somalia was second affected country by anthrax with 44 cases following Ethiopia which reported 542 cases (AU-IBAR 2011)	No diagnostic plan is in place . Treatment is not complex and antibiotics if applied early on with cases of cutaneous and sometimes other forms are curative.
Trypanosomiasis	Trypanosoma brucei	N/A	N/A	Neither treatment nor diagnostic plan is in place
Brucellosis	Brucella species	Prevalence of 0.6% in Southwest State and Benadir region (Hussein, Singh et al. 1978) using slow agglutination microtiter method. Cases of two Somali migrants in the	Seroprevalence survey in Southwest State and Benadir region reported brucella infection in government farms (2.7%), pastoralist livestock (11.9%) (Hussein, Singh et al. 1978) using the slow agglutination microtiter method. Bovine	A National contingency plan for brucellosis developed in 2020

		UK were presented and later investigations confirmed to be brucellosis (Wheat, Dabbs et al. 1995, Javaid, Farrugia et al. 2013)	screening in Benadir and Jubaland States used Milk Ring Test (MRT), Serum Agglutination Test (SAT) and Compliment Fixation Test (CFT) and reported 9.5%, 12.2% and 47% respectively (Wernery, Karani et al. 1976). Another serological investigation on goats slaughtered in Mogadishu abattoir employed Rose Bengal Plate Test (RBPT), SAT, 2-Mercaptoethanol test (2-ME test) and Coombs Antiglobulin Test (AGT) and found 2.8%, 2.8%, 1.6%, and 5.6% respectively (Falade and Hussein 1979). Camel surveys in Puntland State and Somaliland employing a competitive and indirect enzyme-linked immunosorbent assay (ELISA) reported 7% and 3.1% seropositivity respectively (Ghanem, El-Khodery et al. 2009, Mohamud, Kothowa et al. 2021) whereas camels in Galmudug and Hirshabele showed 1.9% and 0.3% positivity using SAT and CFT tests (Baumann and Zessin 1992). Another SAT screening of camels in Benadir and Jubaland State reported an overall 10.4% prevalence (Andreani, Prosperi et al. 1982). Ruminant brucellosis ranged from 7.2% to 19.4% in cattle and 4.4% to 5.3% in sheep respectively using SAT, c-ELISA and RBPT methods (Andreani, Prosperi et al. 1982, Hassan-Kadle, Osman et al. 2021).	

NEXT STEPS AND ACTION PLANS

After finalizing the list of priority zoonotic diseases, workshop participants discussed next steps and action plans to address the priority zoonotic diseases using a multisectoral, One Health approach. Participants were first asked to develop next steps and action plans for how to address the priority diseases using a multisectoral, One Health approach. Participants were then asked to develop specific next steps for their sectors. A summary of the recommendations organized by theme follows:

- Multisectoral, One Health Coordination Mechanisms
- Surveillance
- Laboratory
- Outbreak Response
- Preparedness Planning
- Workforce

Each One Health sector and relevant partners present at the workshop then developed specific next steps for their sector and institution.

APPENDIX A: Overview of the One Health Zoonotic Disease Prioritization Process

HTTPS://WWW.CDC.GOV/ONEHEALTH/GLOBAL-ACTIVITIES/PRIORITIZATION.HTML

Five Steps for CDC's One Health Zoonotic Diseases Prioritization Tool and Workshop

BEFORE THE WORKSHOP

STEP 1

PREPARE FOR THE WORKSHOP

- Contact the CDC One Health Office at least 60 days before the workshop
- Work with in-country leadership to identify 8 to 12 voting members from all relevant sectors to participate in facilitated group work
- Clearly define the purpose and goal of the workshop with all sectors to be represented
- Generate a list of all endemic and/or emerging zoonoses to be considered for ranking; include input from all represented sectors
  - Note: Involves gathering reportable diseases lists

DURING THE WORKSHOP

STEP 2

DEVELOP CRITERIA

- Identify 5 to 8 criteria that will be used to define the relative national importance of the list of zoonoses; criteria should be locally appropriate and agreed upon by voting members

STEP 3

DEVELOP QUESTIONS

- Develop one categorical question for each of the selected criteria

STEP 4

RANK CRITERIA

- Each voting member individually ranks the selected criteria; individual scores are combined to produce an overall ranked list of criteria

STEP 5

PRIORITIZE ZOONOTIC DISEASES

- Score each zoonotic disease based on the answers to the categorical questions for each weighted criterion using the One Health Zoonotic Disease Prioritization Tool
- Discuss next steps for multisectoral engagement for prioritized zoonoses

WORKSHOP OUTCOMES

OUTCOMES

- Prioritized list of at least 5 zoonotic diseases that are agreed upon by all stakeholders at the end of the workshop
- Discussions about next steps for the prioritized zoonoses in terms of identifying areas for multisectoral engagement in developing control and prevention strategies
- Workshop summary that includes the details of the process, the list of prioritized zoonoses, and discussions and recommendations by the participants on how to jointly address capacity building, prevention, and control of prioritized zoonotic diseases
- Final report, approved by all ministries representing core voting members, within six weeks of workshop completion

APPENDIX B: Final Outputs of the One Health Zoonotic Prioritization Tool in Somalia

Disease	Etiologic Agent	Raw Score	Final Score
Brucellosis	Genus Brucella (multiple species)	0.654	0.780
Anthrax	*Bacillus anthracis*	0.814	0.970
Q fever	*Coxiella burnetii*	0.420	0.500
Zoonotic E. coli	*Escherichia coli*	0.350	0.417
Leptospirosis	*Leptospira interrogans*	0.447	0.533
Rickettsiosis	Genus *Rickettsia* (multiple species)	0.364	0.434
Campylobacteriosis	Genus *Campylobacter* (multiple species)	0.511	0.609
Salmonellosis	Several salmonella groups	0.511	0.609
Bovine Tuberculosis	*Mycobacterium bovis*	0.492	0.587
Listeriosis	*Listeria monocytogenes*	0.252	0.301
Rift Valley fever	Rift Valley fever virus	0.839	1.000
Hepatitis E	Hepatitis E virus	0.248	0.295
Crimean-Congo Haemorrhagic fever	Crimean-Congo haemorrhagic fever	0.440	0.525
Camelpox	Camelpox virus	0.332	0.395
Middle East Respiratory Syndrome coronavirus	Middle East respiratory syndrome CoV	0.816	0.973
Rabies	Rabies virus	0.546	0.651
Zoonotic influenza viruses	Avian influenza viruses	0.255	0.303
Chikungunya	Chikungunya virus	0.453	0.540
Dengue Fever	Dengue Fever virus	0.453	0.540
Ebola virus	Ebola virus	0.460	0.548
Yellow fever	Yellow fever virus	0.140	0.167
Orf (contagious ecthyma)	Parapox virus	0.185	0.220
West Nile Fever	West Nile Virus	0.039	0.047
Fascioliasis (Liver fluke)	*Fasciola hepatica* and *Fasciola gigantica*	0.546	0.651
Cysticercosis/Taeniasis	*Taenia saginata*	0.331	0.395
Trypanosomiasis	*Trypanosoma brucei rhodesiense*	0.687	0.819
Echinococcosis (Hydatidosis)	Genus *Echinocococcus*	0.546	0.651
Toxoplasmosis	*Toxoplasma gondii*	0.546	0.651
Schistosomiasis	Only zoonotic *Schistosoma* species	0.398	0.474
Leishmaniasis	Only zoonotic *Leishmania* species	0.393	0.469
Cryptosporidiosis	Genus *Cryptosporidium*	0.296	0.353
Giardiasis	*G. lamblia (duodenalis)*	0.598	0.713
Aspergillosis	Genus *Aspergillus*	0.159	0.190

APPENDIX C: Final Outputs of the One Health Zoonotic Prioritization Tool in Somalia

**Criteria A**	Socioeconomic Impact (including sensitivity)	criterion weight = 0.43
**Question:**	What is the Socio-Economic, political and security impact of the disease?	
**Assumptions:**	minimal (0-1 aspects),<br>low (>1-3),<br>moderate (>3-4),<br>high (>4-5),<br>very high (>5)	
**Answers:**	A. Minimal impact<br>B. Low impact<br>C. Moderate impact<br>D. High impact<br>E. Very high impact	Score = 0<br>Score = 1<br>Score = 2<br>Score = 3<br>Score = 4

**Criteria B**	Environmental Aspects	criterion weight = 0.13
**Question:**	Is increased incidence/Prevalence of the disease and the impact of the environment is associated with extreme weather events?	
**Assumptions:**	drought, flooding, weather events, seasonal pattern, disease impact on Environment	
**Answers:**	A. No<br>B. Weak<br>C. Moderate<br>D. Strong	Score = 0<br>Score = 1<br>Score = 2<br>Score = 3

**Criteria C**	Availability of Intervention (feasibility)	criterion weight **= 0.16**
**Question:**	Are control measures available in the country?	
**Assumptions:**	treatment, Diagnostic capacities, vaccines and other preventive measures	
**Answers:**	Not available for both (Human and Animal)<br>B. Yes available in Human only<br>C. Yes available in Animals only<br>D. Yes available for both (Human and Animal)	Score = 0<br>Score = 1<br>Score = 2<br>Score = 3

**Criteria D**	Burden of disease in humans	criterion weight **= 0.16**
**Question:**	Is there infection prevalence in the Somalia and East Africa region in the last 10 years in humans?	
**Assumptions:**		

**Answers:**	A. No, in both (Somalia and East Africa)<br>B. Yes, in the region (East Africa) only<br>C. Yes, in Somalia only<br>D. Yes, in both (Somalia and East Africa)	Score = 0<br>Score = 1<br>Score = 2<br>Score = 3

**Criteria E**	Burden of disease in animals	criterion weight = **0.12**
**Question:**	Has the disease been detected/occurred in Somalia and in the East Africa region	
**Assumptions:**		
**Answers:**	A. No, in both (Somalia and East Africa)<br>B. Yes, in the region (East Africa) only<br>C. Yes, in Somalia only<br>D. Yes, in both (Somalia and East Africa)	Score = 0<br>Score = 1<br>Score = 2<br>Score = 3

APPENDIX D: Next steps and action plans

Strengthen laboratory systems and networks to ensure early detection

Proposed activities	2023	2024	2025	2026	2027	2028	Main responsible sectors	Potential /proposed collaborators
Capacity building for veterinary and health workers in the diagnosis of priority zoonoses							MOH, MoLFR,	Academics, WHO, WOAH, CDC, FAO
Conduct assessments of laboratories at all administrative levels inclusive of needed capacities (e.g. biosafety/biosecurity, international standards) for priority zoonotic diseases.							MOH, MoLFR,	Academics, WHO, WOAH, CDC, FAO
Evaluate the required equipment is available, functioning and calibrated for running of each laboratory							MOH, MoLFR	Academics, WHO, WOAH, CDC, FAO
Establish a national veterinary and public health reference laboratory with diagnostic capacity for priority zoonotic diseases, inclusive of proficiency testing and standardized test protocols at all levels							MOH, MoLFR,	Academics, WHO, WOAH, CDC, FAO
Ensure diagnostic capacity for select priority zoonotic diseases isstate-level state level public health and veterinary laboratories,							MOH, MoLFR,	Academics, WHO, WOAH, CDC, FAO
Develop a laboratory monitoring checklist to ensure all the above activities are taking place and on track.							MOH, MoLFR,	Academics, WHO, WOAH, CDC, FAO
Establish a multisectoral laboratory working group and network of human, animal, and environmental health laboratory experts for developing standardized testing, SOPs and reporting of the priority zoonotic diseases. Laboratories included process samples from people, animals, the environment, vectors, food and toxins, and can represent central and state levels as well as academic							MOH, MoLFR,	Academics, WHO, WOAH, CDC, FAO

and private laboratories participating in the national surveillance system.								
Establishment of quality management system for national veterinary and public health laboratory							MOH, MoLFR,	Academics, WHO, WOAH, CDC, FAO
Accreditations of the laboratory test methods							MOH, MoLFR,	Academics, WHO, WOAH, CDC, FAO
Establish biological specimen referral systems to support coordinated surveillance and outbreak response of priority zoonotic diseases							MOH, MoLFR,	Academics, WHO, WOAH, CDC, FAO
Waste management system in veterinary and human laboratory							MOH, MoLFR,	Academics, WHO, WOAH, CDC, FAO
Develop a Risk management system manuals							MOH, MoLFR,	Academics, WHO, WOAH, CDC, FAO
Conduct a research on about the status of avian influenza, MERS-cov, RVF at country level							MOH, MoLFR,	Academics, WHO, WOAH, CDC, FAO

Preparedness Plan Activity

	2023	2024	2025	2026	2027
Establish multisectoral and multidisciplinary collaboration teams					
Establishment of communication and coordination mechanism (Establish communication between OH researchers)					
Allocation of resource for OH activities					
Capacity building (develop OH training materials)					
Develop awareness raising OH program					
Establish ZD data sharing and information platform					
Conduct a joint risk assessment for priority zoonotic diseases					
Establishment of early warning system (develop tool for data collection and report format, determine frequency of reporting)					
Conduct simulation exercises in the country and across the border					
Determine the baseline level of risk for cross-border spread of zoonotic diseases.					
Develop OH contingency and strategic plan for priority ZD and establishment of OH joint action plan					
Strengthen OH policy and legal framework					
Establish of Institutional collaborative OH research (establish OH research agenda)					

Workforce development

Activity	Sectors	2023	2024	2025	2026	2027	2028	Level of implementation
Conduct a workforce analysis/needs assessment of Human resources including One Health work force gaps analysis.	Human Health, Environment, Animal Health, Plant.							Not Started
Establish a multi-sectoral Taskforce at Federal and Federal members' states level to review the recommendation on workforce analysis/needs identified.	Human Health, Environment, Animal Health, Plant.							Not Started
Establish a workforce surge/database for rapid response and deployment at Federal and Federal members' states level to respond to one health needs.	Human Health, Environment, Animal Health, Plant.							Not Started
Establish a multi-sectoral structure that links the communities and the workforce and the taskforce in addressing the one health needs.	Sub-National Community, Human Health, Environment, Animal Health, Plant.							Not Started
Strengthen workforce coordination, capacity building for all sectors working with communities.	Sub-National Community, Human Health, Environment, Animal Health, Plant.							Not Started
Identify team leads focal points in the sectors to work closely on workforce issues at Federal and Members state level.	Human Health, Environment, Animal Health, Plant.							Not Started
Specify workforce teams and units to work under Rapid response to needs for One Health.	Human Health, Environment, Animal Health, Plant.							Not Started
Work universities and tertiary schools on workforce development with focus on One Health Training.	Human Health, Environment, Animal Health, Plant.							Not Started

Multi Sectoral Approach

Activity	Sectors	2023	2024	2025	2026	2027	2028	Level of implementation
Conduct a multi sectoral coordination workshop to iron out areas of collaboration (Risk communication, Laboratory, Joint Surveillance, Workforce, resource mobilization and Community approach/Sub-national Mechanisms).	Human Health, Environment, Animal Health, Plant.							
Carryout a bridging activity/Workshop to bridge the gaps in Human Health, Environment, Animal Health, Plant.	Human Health, Environment, Animal Health, Plant.							
Establish an independent coordination platform for One health to spearhead One health activities. - A National Agency. (lead by President/Prime minister level) – Through Act of parliament.	Human Health, Environment, Animal Health, Plant.							
Carryout Multi-sectoral sessions to strengthen One Health aspects in the country.	Sub-National Community, Human Health, Environment, Animal Health, Plant.							
Strengthen cross border collaboration and harmonization to address One Health issues jointly.	Sub-National Community, Human Health, Environment, Animal Health, Plant.							

Surveillance

Planned activities	2023	2024	2025	2026	2027	Level of implementation	Main authority	Potential Collaborators
To strengthen and expand capacity for Early warning system for one health approach						All levels	MOH, ministry of livestock,	WHO, FAO, VSf-Swiss, university of Liverpool, ILRI and ICIPE, CDC Africa
Strengthen capacity for epidemiologic data collection, analysis, cross border surveillance and dissemination to all partners through joint periodic information products						All levels	MoH, ministry of livestock, ministry of agriculture	WHO, FAO, VSf-Swiss, university of Liverpool, ILRI, ICIPE, CDC
To strength capacity for timely reporting of zoonosis to health partners						National	MoH, ministry of livestock	WHO, FAO, VSf-Swiss, university of Liverpool, ILRI, ICIPE, CDC
Strengthen capacity for joint risk assessment to identify vulnerable populations to zoonosis						All levels	MoH, ministry of livestock,	WHO, FAO, VSf-Swiss, university of Liverpool, ILRI, ICIPE, CDC
To strengthen and expand integrated rapid response teams for investigation of zoonosis						District level	MoH, ministry of livestock,	WHO, FAO, VSf-Swiss, university of Liverpool, ILRI, ICIPE, CDC
To strengthen capacity for Risk communication and community engagement						Community level	Ministry of health, ministry of livestock, Ministry communication	WHO, FAO, VSf-Swiss, university of Liverpool, ILRI, ICIPE, CDC
To strengthen capacity for the prevention and control of zoonosis in different population groups						Health facility and community level	MoH, ministry of livestock	WHO, FAO, VSf-Swiss, university of Liverpool, ILRI, ICIPE, CDC Africa

References

1. Carbone, F. and G. Accordi, *The Indian Ocean coast of Somalia.* Marine Pollution Bulletin, 2000. **41**(1-6): p. 141-159.
2. Maystadt, J.-F. and O. Ecker, *Extreme weather and civil war: Does drought fuel conflict in Somalia through livestock price shocks?* American Journal of Agricultural Economics, 2014. **96**(4): p. 1157-1182.
3. Samatar, A.I., *Structural adjustment as development strategy? Bananas, boom, and poverty in Somalia.* Economic Geography, 1993. **69**(1): p. 25-43.
4. Shuriye, A. *The Prospective Economic Cooperation between Somalia and Some ASEAN Nations. Proceedings 2022, 82, 3*. in *International Academic Symposium of Social Science 2022*. 2022. s Note: MDPI stays neutral with regard to jurisdictional claims in published ….
5. Conan, S.-H. and G. Brummer, *Fluxes of planktic foraminifera in response to monsoonal upwelling on the Somalia Basin margin.* Deep Sea Research Part II: Topical Studies in Oceanography, 2000. **47**(9-11): p. 2207-2227.
6. Warsame, A.A., et al., *Climate change and crop production nexus in Somalia: an empirical evidence from ARDL technique.* Environmental Science and Pollution Research, 2021. **28**(16): p. 19838-19850.
7. Griffiths, C.L., *Coastal marine biodiversity in East Africa.* 2005.
8. Amir, O.G., *Wildlife trade in Somalia.* Report to the IUCN/SSC Antelope Specialist Group, 2006. **28**.
9. Amir, O.G. *The silent victim: The wildlife of Somalia and its rate of extiction*. in *Proceedings of EASS/SSIA International Congress of Somali Studies. Variations on the theme of Somaliness*. 2001. Centre for Continuing Education, Åbo Akademi University.
10. Farah, Z., et al., *Camel dairy in Somalia: Limiting factors and development potential.* Livestock Science, 2007. **110**(1-2): p. 187-191.
11. Hussein, B.M., *The Evidence of toxic and radioactive wastes dumping in Somalia and its impact on the enjoyment of human rights: a case study.* Unite d Nations Human Rights Council, 2010.
12. Sumaila, U.R. and M. Bawumia, *Fisheries, ecosystem justice and piracy: A case study of Somalia.* Fisheries Research, 2014. **157**: p. 154-163.
13. Orloff, K. and H. Falk, *An international perspective on hazardous waste practices.* International Journal of Hygiene and Environmental Health, 2003. **206**(4-5): p. 291-302.
14. Hägerdal, N., *Toxic waste dumping in conflict zones: Evidence from 1980s Lebanon.* Mediterranean Politics, 2021. **26**(2): p. 198-218.
15. Seal, A.J., et al., *Use of verbal autopsy for establishing causes of child mortality in camps for internally displaced people in Mogadishu, Somalia: a population-based, prospective, cohort study.* The Lancet Global Health, 2021. **9**(9): p. e1286-e1295.
16. Thalheimer, L., M.P. Schwarz, and F. Pretis, *Large weather and conflict effects on internal displacement in Somalia with little evidence of feedback onto conflict.* Global Environmental Change, 2023. **79**: p. 102641.
17. Sinclair, J.R., *Importance of a One Health approach in advancing global health security and the Sustainable Development Goals.* Revue scientifique et technique (International Office of Epizootics), 2019. **38**(1): p. 145-154.

***
